# Supplementary material for: Effective Blue Light-Absorbing AuAg Nanoparticles in InP Quantum Dots-Based Color Conversion
Source: Materials (Basel). 2022 Nov 27;15(23):8455. doi: 10.3390/ma15238455 (PMC9736556; doi:10.3390/ma15238455)
Supplement: Supplementary file 1 [file materials-15-08455-s001.zip › materials-1996698-supplementary.pdf]

Supplementary Materials

Effective Blue Light-Absorbing AuAg Nanoparticles in InP Quantum Dots-Based Color Conversion

Hyo-Jin Yeo [1,†], Suk-Young Yoon [1,†], Dae-Yeon Jo [1], Hyun-Min Kim [1], Jeonghun Kwak [2,*], Sung-Phil Kim [3], Myung-Joon Kim [4] and Heesun Yang [1,*]

[1] Department of Materials Science and Engineering, Hongik University, Seoul 04066, Republic of Korea
[2] Department of Electrical and Computer Engineering, Seoul National University, Seoul 08826, Republic of Korea
[3] Department of Biomedical Engineering, Ulsan National Institute of Science and Technology, Ulsan 44919, Republic of Korea
[4] Department of Big Data Application, Hannam University, Daejeon 34430, Republic of Korea
* Correspondence: jkwak@snu.ac.kr (J.K.); hyang@hongik.ac.kr (H.Y.)
† These authors contributed equally to this work.

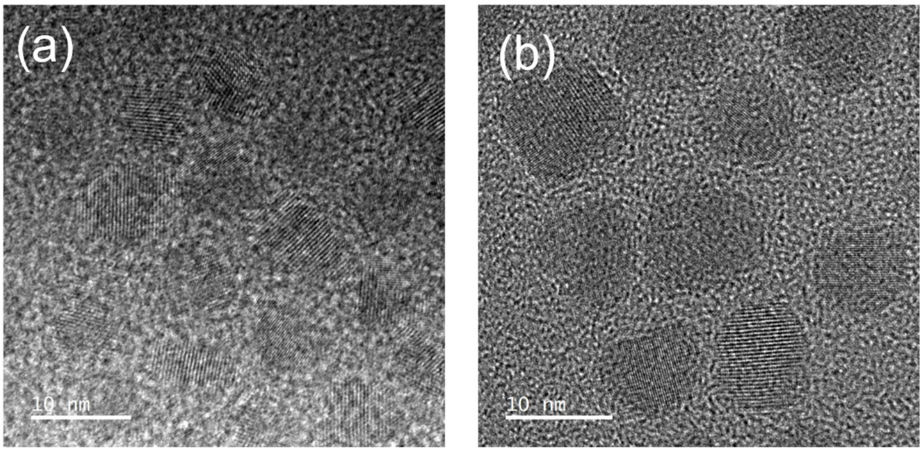

Figure S1. Higher-magnification TEM images of (a) green and (b) red InP/ZnSe/ZnS QDs (scale bars are 10 nm for both).

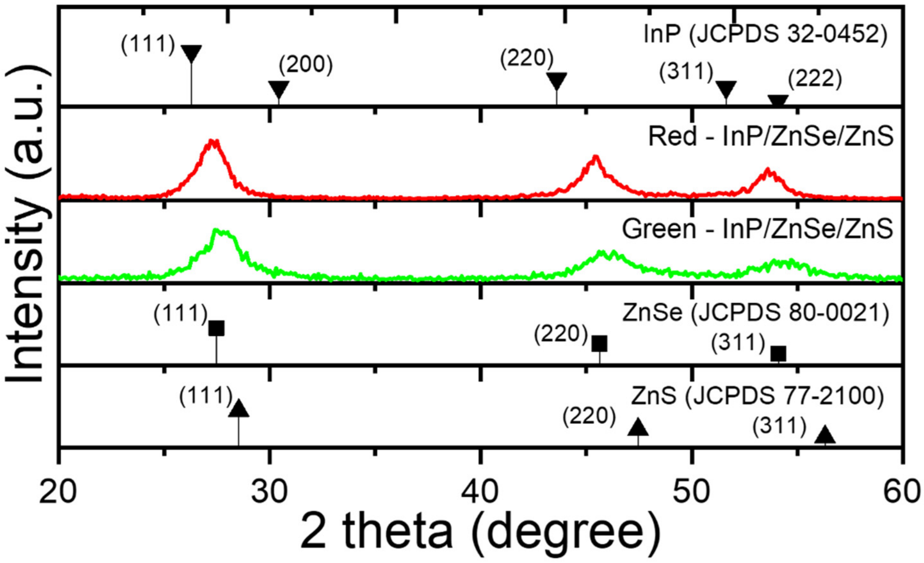

Figure S2. Powder XRD patterns of green and red InP/ZnSe/ZnS QDs.

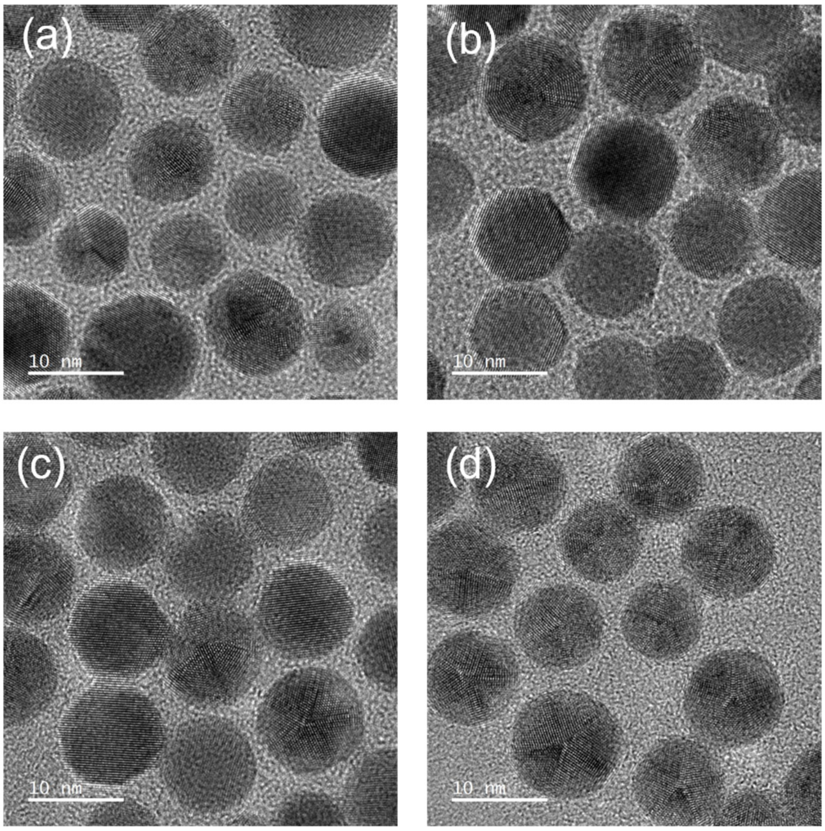

Figure S3. Higher-magnification TEM images of AuAg alloy NPs synthesized with Au/Ag precursor ratios of (a) 0.45, (b) 1, (c) 2.3, and (d) 3 (scale bars are 10 nm for all).

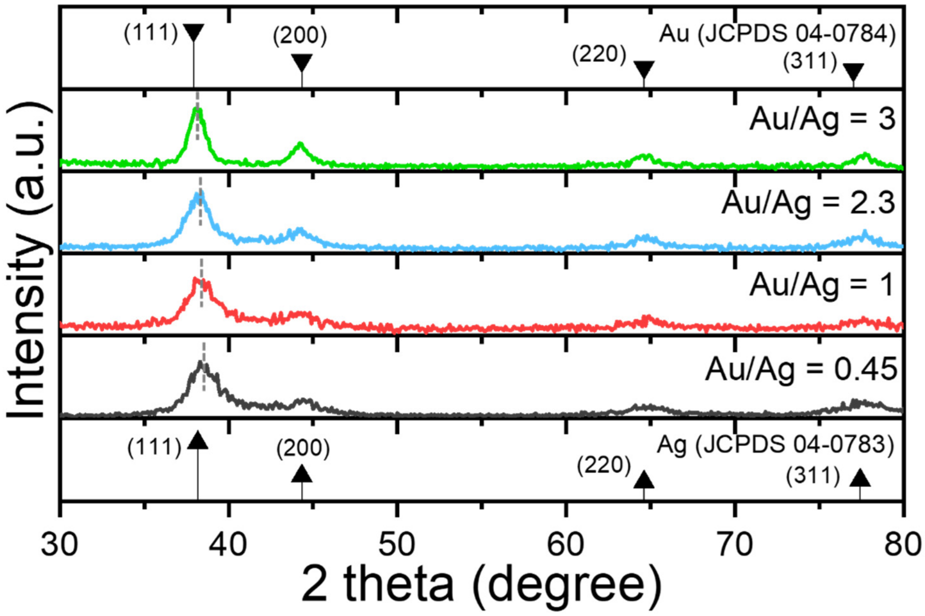

Figure S4. XRD patterns of a series of AuAg NPs with different Au/Ag ratios.

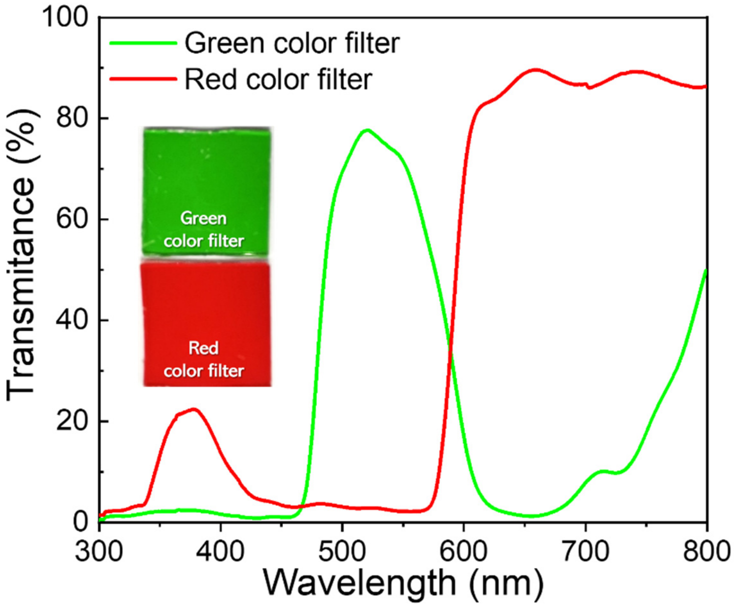

Figure S5. Typical transmittance spectra of commercial LCD green and red color filters.
